# Screening Blood Donors at Risk for Malaria: Reply to Hänscheid et al.

**DOI:** 10.3201/eid0808.020200

**Published:** 2002-08

**Authors:** A. Benito, J.M. Rubio

**Affiliations:** Institute of Health Carlos III, Madrid, Spain

**To the Editor:** The letter to editor by Hänscheid et al. addresses our suggestion that polymerase chain reaction (PCR) could serve as a reference test for screening blood donations. At present, PCR is the most sensitive and specific method for parasite detection in malaria-endemic areas. However, additional measures should be taken into account, such as serologic testing, refining donor history, defining at-risk locations, and delimiting malaria-endemic areas. Therefore, we do not suggest that only PCR should be used as a reference method to exclude blood donors at risk, but it could help shorten the deferral period for blood donor (currently 3 years after an asymptomatic person leaves the malaria-endemic area). PCR should be accompanied by serologic tests and the elimination of actual or possible plasmodial infection in the blood donor.

In our laboratory, we use the indirect fluorescent antibody test (IFAT) for antigens of the four plasmodia species, in addition to PCR screening. At present, we have analyzed a total of 531 blood samples (406 more than the 125 described in our previous letter to the editor) from possible donors at risk for malaria, and only the five described in the letter were malaria positive. Moreover, 40% (50 of 125) of these sera were negative by IFAT (unpub. data), a fact that indicates the importance of having a complete donor history and being certain of the patient’s origin in the context of malaria endemicity (i.e., several geographic areas without malaria transmission in some Central and South American countries could be excluded as malaria-risk areas; these areas coincide with sera negative by IFAT).

On the other hand, with a standard 450-mL blood donation, parasitemias <90 could test negative by PCR; but, as previously described, this technique should be accompanied by careful questioning, serologic testing, and eliminating parasites from the recipient during blood processing and storage.

We take for granted that, theoretically, any method would have to detect a single parasite per unit of blood to be safe and that little is known about the frequency of low parasitemias. In nature, and in accordance with the parasitologic definition of equilibrium between parasite and host (defined over thousands or millions of years according to different phylogenetic theories), one of the main strategies for parasite survival is sustained malaria transmission, which allows low parasitemias to be ingested by the anopheline vector (the amount of blood ingested by the female anopheline varies from 1.3 to 3.0 µL) (1). In this way, the amount of blood should have sufficient parasites to continue the cycle inside the vector. This fact explains the stability of malaria transmission during dry seasons. Plamodium falciparum infections can persist for at least 1 year in a substantial proportion (10%) of the host (2).

In two-thirds of the cases cited by Mungai et al. (3), the donor-screening process failed, illustrating the difficulties in obtaining accurate travel and immigration histories from donors. In this paper, serologic tests were positive retrospectively in 98% of tested donors, indicating that serologic tests should be a useful screening technique for malaria blood donors; 35% showed parasitemia in blood smears, a level that would have increased if the blood had been analyzed on the day of transfusion and not in retrospective study (after the degradation or deformation of the parasites or loss of staining of parasite chromatin).

Moreover, two of the three cases described by Slinger et al. (4) were in blood donors positive by microscopy or PCR (the other potential blood donor was not available for follow-up). These results show that parasites could have been detected in these cases, reflecting that all blood samples had detectable parasitemias.

Serologic tests in Spain indicate that approximately 50% of the referral donations could be used in transfusion, and travel histories should distinguish the specific destinations or the level of malaria transmission in the area. Most histories are based on the wide areas of transmission listed in travel guidelines. These data should decrease the cost of testing per blood donation when we add the value of the blood donation to the real cost of death prevented.

In conclusion, our initial results, now accompanied by serologic results, justify the exclusion criteria we first reported (5). Screening tests for blood donors (e.g., PCR) should be used as a reference technique that could shorten the deferral period for blood donors. In all well-reported cases (complete studies with follow-up) of transfusion-associated malaria described in Canada and the United States, PCR could have detected the parasites in blood.

Finally, the study of donors at risk could serve as indirect surveillance for asymptomatic infections and could play an important role in detecting autochthonous malaria transmission in the United States (5) or Spain where local anopheline vectors exist. An additional benefit for parasite detection is that it would permit the donor to be treated and locally acquired malaria to be eliminated. The Anopheline mosquito vectors of malaria still exist in the United States at levels sufficient to sustain malaria transmission, and dozens of cases of autochthonous malaria transmission have been reported in the United States over the past 15 years (6).
